# The GALENOS approach to triangulating evidence: a structured approach for integrating information from human and animal studies

**DOI:** 10.1186/s12874-026-02891-4

**Published:** 2026-06-02

**Authors:** Thomy Tonia, Julian P.T. Higgins, Virginia Chiocchia, James Downs, Emily Wheeler, Jennifer Potts, Katharine A. Smith, Malcolm Macleod, Gin S. Malhi, Andrea Cipriani, Georgia Salanti

**Affiliations:** 1 Institute of Social and Preventive Medicine, University of Bern, Bern, Switzerland; 2 Population Health Sciences, Bristol Medical School, University of Bristol, Bristol, UK; 3 MQ Mental Health Research, London, UK; 4 Department of Psychiatry, University of Oxford, Oxford, UK; 5 Oxford Precision Psychiatry Lab, NIHR Oxford Health Biomedical Research Centre, Oxford, UK; 6 NIHR Oxford Health Biomedical Research Centre, Oxford, United Kingdom; 7 Centre for Clinical Brain Sciences, University of Edinburgh, Edinburgh, UK; 8 Academic Department of Psychiatry, Kolling Institute, Northern Clinical School, Faculty of Medicine and Health, The University of Sydney, Sydney, Australia

**Keywords:** Triangulation, Mental health, Living systematic review, Co-production, Evidence synthesis, Risk of bias, Triangulation meeting

## Abstract

**Background:**

Most decision-making research involves collating multiple types of evidence, and such integration should be a core component of investigative practice in science. Triangulation is one formal approach that has proven effective, typically drawing on sources of evidence with different biases. However, there remains no standardised method for its implementation and no clear guidance on how to apply triangulation in research contexts—particularly when synthesising evidence across human and animal data.

**Methods:**

GALENOS (Global Alliance for Living Evidence on aNxiety, depressiOn and pSychosis) has introduced GATE (GALENOS Approach to Triangulating Evidence), a multifaceted framework for such triangulation. To further develop GATE, we here systematically searched the literature for work describing triangulation processes for human and animal data.

**Results:**

As a key component of GATE, we provide conceptual details and practical guidance on how to prepare, organise, conduct, and follow up on triangulation meetings.

**Conclusions:**

Based on findings from this literature and our experience with GATE, we updated our approach.

**Supplementary Information:**

The online version contains supplementary material available at 10.1186/s12874-026-02891-4.

## Background

Research triangulation is a process in which multiple sources of different evidence addressing the same question are integrated through collection, examination, comparison, and interpretation [1]. Triangulation can be used to test hypotheses and understand phenomena with increased rigor than can be achieved by considering a single source [2–5]. The triangulation process evaluates the potential biases and their direction in each evidence strand, and, if biases are unrelated to each other, weaves the evidence strands to reach a conclusion [6]. Triangulation has been successfully used in social sciences and education research, with several examples from the public health field and epidemiology [3, 7].

Although much research entails some sort of evidence triangulation, the process is often informal, not sufficiently structured, nor is it fully transparent or clearly documented. Previous attempts to structure the triangulation processes have described it as a stepwise approach that includes (up to) 12 steps grouped broadly into three main categories: (a) planning the triangulation process, (b) conducting the triangulation of evidence and analysing the data, and (c) communicating the results or action points [1, 4]. The process may also include a “triangulation meeting”, where stakeholders involved in the process present and evaluate the whole body of evidence, alter, or modify the starting research hypotheses and identify the need for additional data [1]. The role of a triangulation meeting shares elements with meetings to develop clinical recommendations. There is a lot of guidance about the development of clinical recommendations, with clear instructions about how to structure and hold meetings [8–10] and generate quality checklists and reviews [11, 12]. However, detailed theoretical, methodological, and operational guidance on how to plan, conduct, and evaluate triangulation is still lacking [3, 6]. We aim to fill this gap as part of our collective research endeavours of the GALENOS project (Global Alliance for Living Evidence on aNxiety, depressiOn and pSychosis) [13].

GALENOS is a Wellcome-funded project that carries out a series of living systematic reviews (LSRs) on the mechanisms, prevention, and treatment of anxiety, depression, and psychosis [14]. People with lived experience of mental health issues are involved in all steps of GALENOS, and the project aims to inform priority setting for research conducted to develop new treatments or diagnostic approaches [15]. In our experience, the involvement of people with lived experience makes a critical contribution to the success of, and value which can be derived from, the process of research priority setting that meets identified unmet needs. All GALENOS LSRs aim to include different types of evidence, including data human and non-human animal studies (referred to from now on as “animal studies” for brevity). Triangulation of these two evidence theses, and consideration of their different biases, as well as the integration of lived experience, leads to recommendations for future research [15]. The proof-of-concept approach, GATE (GALENOS Approach to Triangulating Evidence), has been developed in the first three years of the project. It describes the collection, evaluation and integration of different types of evidence using a multi-disciplinary and co-produced approach. More details on GATE can be found elsewhere [16].

As part of the living nature of GALENOS and to further develop the GATE process, we set out to (a) summarise the literature about the process of triangulation of evidence from animal and human data; and (b) elaborate and update GATE with an emphasis on the triangulation practicalities and processes, in order to make its implementation in triangulation meetings structured, transparent and reproducible.

## Methods

In developing our proposal for a structured approach for triangulation meetings, we draw on our experience from GATE [16], applied so far in three triangulation meetings; on existing guidance on how to structure guideline panel recommendation meetings [8–10, 17–19]; and on the results of the literature review described below [20–23].

We conducted two searches: a systematic search using the terms “triangulat*” and “animal” or “in vivo” in the title and abstract fields until March 2025. We searched PubMed and included any study that clearly triangulated evidence from human and animal data or provided a framework for doing so. One reviewer (TT) screened titles and abstracts, and two reviewers (VC and TT) screened full texts. Any disagreements were resolved by discussion.

For the second search, we searched PubMed and Google for “triangulation meeting“[Title/Abstract:~3] OR “triangulation workshop“[Title/Abstract:~3] to identify any documentation of triangulation meetings. We included any published paper that described holding a meeting or a workshop, which had as a main aim to triangulate data (not restricted to animal and human data triangulation).

## Results

### Literature review and experience from GALENOS triangulation meetings

The first search identified 11 documents that described some kind of data triangulation from human and animal studies [20, 21, 24–32]. Only two of these papers [20, 21] described a more structured triangulation process. Using these two papers, we identified two additional documents providing some more information on the practicalities of how the US National Toxicology Program (NTP) and the International Agency for Research on Cancer (IARC) triangulate evidence [22, 23]. Our second search for documented triangulation meetings identified 13 papers [1, 2, 15, 33–42], but none of them provided practical information on how triangulation meetings were conducted, apart from the one describing a meeting of GALENOS [15]. Stemming from the above, only four papers [20–23] provided some input into the development of our practical processes for triangulation processes and meetings. This input was very general and only slightly touched upon subjects such as procedures, agenda, participants, timeline and content. Full information on the literature review and study selection process can be found in the Additional file 1 (Details on the literature searches), as well as a table with the characteristics of the 11 papers that described a triangulation process of animal and human data (Table A, Additional file 1).

**Fig. 1:**
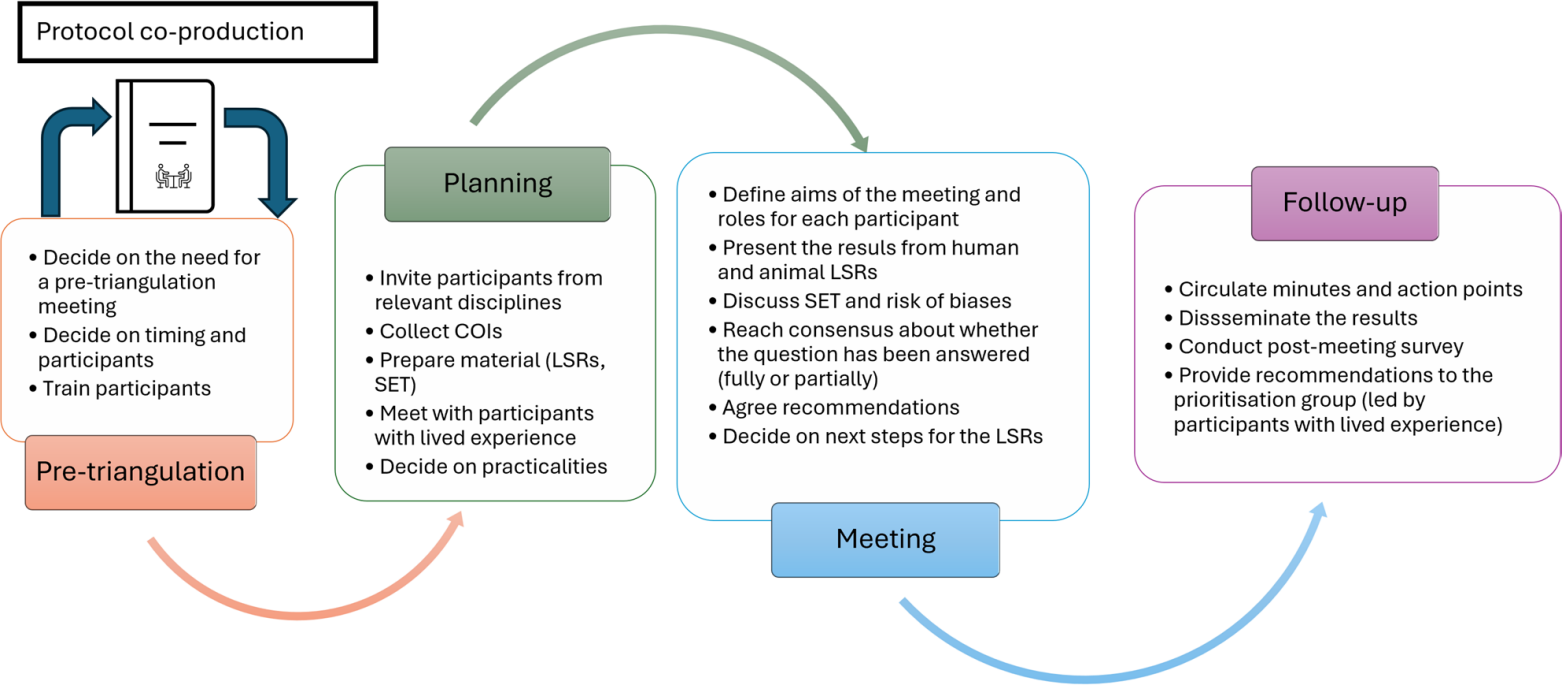
The steps in GALENOS Approach to Triangulation Evidence (GATE).COIs: Conflicts Of Interest, LSRs: Living Systematic Reviews; SET: Summary of Evidence tables

So far, for GALENOS, we have held three triangulation meetings. At the time of the literature search, only one of the triangulation meetings has been described in a publication [15]. Based on the contents of the documents identified in the literature, further deliberations and feedback within GALENOS, and established guidance about how to hold meetings for clinical guidelines development [8–10, 17–19], we suggest the following structure for triangulation exercises (Fig. 1).

Consider that a clear research question has been set, and the standard steps of the LSRs are followed to answer it: data are collected according to predefined protocols, and then synthesised (separately for animal and human studies, and by study design) using state-of-the-art methods [43]. The methodological framework for producing and evaluating evidence using LSRs of different types of data, such as human and animal data will be illustrated in another paper, where we discuss state-of-the-art meta-analytic methods for different types of data, and how to create evidence summaries that can inform a triangulation process [44]. Triangulation considerations should be made alongside the LSR development process, starting with the drafting of the protocol.

#### Pre-triangulation meetings

##### Decide if a pre-triangulation meeting is needed

In GALENOS, the protocol of the systematic review is co-produced involving people who represent all the relevant disciplines required to answer the starting research question, including experts with lived experience. Depending on the complexity of the subject, the author team might decide that a *pre*-triangulation meeting is needed to refine and discuss some aspects of the protocol more efficiently, potentially including external independent experts in the discussion (See Box A, Additional file 1 for examples from one of our LSRs on circadian disruption and mood disorders). A pre-triangulation meeting is usually warranted when:

- agreement of the PICO framework to be used is not unanimous;
- the field is either completely novel, or it is so broad that some focussing is required to make it manageable and meaningful;
- the relevant study designs needed to address a question are not immediately self-evident, and further discussion between content experts and methodologists is needed.

The timing of a pre-triangulation meeting is important. It cannot be too early in the process, as some background work on the protocol must be done beforehand. The pre-triangulation meeting is not intended to write the protocol but rather to refine it, focus it, or resolve conflicts. The meeting should also not be held too late: it is important to minimise influence from the review results and to ensure that resources spent on data collection are used efficiently. For example, if findings are ultimately deemed irrelevant, the time and effort invested would have been unnecessary. Therefore, we recommend that any preliminary data and results be shown at the pre-triangulation meeting in a completely blinded fashion, i.e. without indicating whether or not a particular hypothesis is supported. Moreover, any decisions taken at the pre-triangulation meeting and their rationale should be defined in subsequent versions of the protocol and transparently reported as amendments to the original protocol.

Ideally, the experts who attend the pre-triangulation meetings should also attend the subsequent main triangulation meeting(s).

##### Training for the pre-triangulation panel

Several of the aspects below could apply to triangulation meetings as well.

While the panel comprises experts from many different domains, successful triangulation depends on all participants having sufficient familiarity with each other’s areas to communicate effectively and “speak a common language.”

In the latest GATE application, we started with a half-day- workshop that included presentations on topics related to methodological issues of systematic reviews of animal and human studies, general discussion on biases in the different study designs relevant to the question, a presentation of co-production by persons with lived experience, and training on triangulation methodology (Table 1).

**Table 1:** A checklist of training topics for the triangulation panel members

Training topics	Details
Clinical scientific context of the research question	Clarifying the research question – defining exposures, outcomes, populations, and mechanisms of interest. Overview of animal models – common models used for the outcomes of interest, their strengths and limitations. Relevant human study designs – randomised controlled trials, cohort studies, case-control studies, cross-sectional studies, and their role in causal inference.
Methodological issues on SRs	Synthesis approaches – narrative synthesis, quantitative meta-analysis, and challenges in synthesis of animal studies Heterogeneity – study design differences, outcome measures, and differences between human and animal meta-analyses Updating and living systematic reviews – approaches for iterative evidence incorporation.
Biases in the evidence	Biases in human data – study design limitations, measurement error, selective reporting. Biases in animal data – internal validity, external validity, translational limitations, and limited reporting of experimental detail. Risk of bias tools by design– e.g., Risk-of-bias tool for randomised trials (RoB 2) [45], Risk Of Bias In Non-randomised Studies - of Interventions (ROBINS-I) [46], SYstematic Review Centre for Laboratory animal Experimentation Risk of Bias tool (SYRCLE RoB tool) [47]
Co-production	Roles and responsibilities – clarified through governance documents. Onboarding materials – induction packs, ongoing training, and access to curated resources. Collaborative decision-making – consensus-building strategies for multidisciplinary teams.
Training on triangulation methodology	What is triangulation? – definitions and theoretical basis. How triangulation works – formal frameworks and approaches (convergence, complementarity, dissonance). Cross-species translation – challenges in extrapolating evidence from animals to humans.

This training supported knowledge exchange and facilitated communication among all participants [48], enabling the discussions during the pre-triangulation and triangulation meetings to be more effective.

### Planning the triangulation meeting

Several of the aspects below could apply to pre-triangulation meetings as well.

#### Decide who will be in the triangulation panel

Comprehensive evaluation of different evidence sources and their biases can be performed only when all relevant disciplines and perspectives are involved in the triangulation process [49]. This is especially the case for public health questions [50]. GATE triangulation panels include mental health clinicians, experts in human and animal research, epidemiologists, people with lived experience, and methodologists (Table 2).

**Table 2:** A checklist for planning a triangulation meeting

Set up the triangulation panel	Gender balanced, global, and includes participants with lived experience. Multidisciplinary (in GATE, we include participants with expertise in clinical psychiatry, clinical studies, preclinical studies, research methodology, evidence synthesis, statistical analysis and lived experience). Co-chaired across disciplines (for example, by people with experience in evidence synthesis and triangulation and people with lived experience).
Distribute materials prior to the meeting	Slides of the key methodology followed to produce the evidence. Summary of evidence tables for the animal and human studies, including risk of bias assessments.
Organise the meeting agenda	Presentation and discussion of the summary of evidence tables. Assessment of whether the sources of evidence (for example, from animal and human studies and possibly the different study designs) are in agreement.
Create and share the meeting output	Assessment of whether the starting research question has been answered. Recommendations for future work and research prioritisation. Recommendations for the next iteration of the living systematic review.

These experts are identified through the GALENOS International Advisory Board and the network of personal contacts of the GALENOS investigators. Equally important, the work in GALENOS is co-produced with people with lived experience (identified via the Global Lived Experience Advisory Board (GLEAB) of GALENOS) [15, 51], who are essential participants in the triangulation process. The following roles should be represented:

- *Chair(s)*: As with any meeting, the Chair ensures a smooth process, that all voices are equally heard, and that all steps of the triangulation process are followed. The Chair(s) of such meetings should have experience in evidence-based medicine, methodology, and ideally, triangulation efforts. Clinical experience on the topic is not necessary but could be useful if available. If a series of meetings is planned (e.g. pre-triangulation and triangulation meetings), we suggest having the same Chair(s) to ensure consistency in the process. To create the necessary space for co-production, the two previous GALENOS triangulation meetings had two co-Chairs, one with methodology expertise and one with expertise through lived experience, with a broad background in mental health science and GALENOS [51]. When there is more than one Chair, it is important for the Chairs to meet beforehand to prepare and establish who will be leading when and what support will be needed.
- *Researchers presenting the evidence*: People who are responsible for gathering the data for animal and human LSRs should present the results, their initial conclusions, and how these relate to the original hypotheses and to answer questions. People who have the general methodological overview of the project should also be present to answer questions when needed. However, representatives of the evidence and methodology experts should not take an active part in the triangulation process itself.
- *External clinical experts*: People with clinical experience in the subject discussed but who have not been involved in the collection/analyses of the data for the LSRs.
- *Experts in human and animal studies*: people who have experience in conducting animal or human research on the subject and have not been directly involved in the LSRs.
- *Methodology experts*: People experienced in evidence synthesis, bias, and triangulation methodology, and who are external to the specific project.
- *People with lived experience*: As a result from our experience of triangulation in GALENOS, we would not embark on a triangulation meeting without the involvement of people with lived experience, and we believe this should be the case in all triangulation meetings where the research question has any relevance whatsoever to human health. In GATE, they are supported by staff from MQ Mental Health Research, a leading global mental health research charity that champions patient and public involvement [52], who are also present at the meetings. At previous triangulation meetings, we had an average of three participants with lived experience. For future meetings, we aim to have a mix of people with lived experience that are specific to the topic being addressed, alongside others who are familiar with the process of triangulation and can facilitate involvement.

#### Collect conflict of interest (COI) forms of everyone involved in the meeting

Everyone’s COI should be shared with all the participants before the meeting. Examples of relevant COI could include being directly involved in the primary studies identified in the systematic reviews or having any vested interests in the selected topic.

#### Prepare summaries of the evidence and send them to participants prior to the meeting

The material gathered for each research question can be lengthy and sometimes complex to understand. In GATE, each LSR produces Summary of Evidence Tables (SET) for human and animal evidence separately, which constitute the main subject of discussion in a triangulation meeting. SET summarise the results of the meta-analyses (if applicable) using numerical and graphical tools and present preliminary judgments of different sources and direction of biases [44]. To facilitate the interpretation of the SET , additional details and explanations should be provided in extended/additional reports of the LSR.

The material should be sent together with the agenda to the panel well in advance of the triangulation meeting. The timing would depend on the length and complexity of the evidence. This material should be reviewed before circulation by people with lived experience, who could suggest edits to the presentation that could make it easier to understand.

For the first triangulation meeting where GATE was applied, we recorded video presentations of the main results and the SET, and we found them useful in engaging participants to consider the material on time [15] (Table 2). Ahead of the triangulation meeting, separate meetings were also held between people with lived experience and the Director of GALENOS to ensure that people with lived experience could ask questions or request clarifications on the evidence presented or terminology used [15]. People with lived experience were also offered specific training on systematic reviews/ evidence essentials by Cochrane. Before and during the triangulation meeting, parallel discussions with people with lived experience can also be held to ensure adequate understanding and that any edits proposed for clarity are considered.

#### Duration and format of the meeting and practicalities

Optimal meeting duration depends on the specifics of each triangulation effort. For GALENOS, we held triangulation meetings over either one full day or two half days. The latter option was chosen to avoid information overload and loss of focus, and to allow more time for training, reflection, and discussion. The triangulation meetings might be more efficient in person or at least in hybrid mode. As the discussions may be long, complex and focused on understanding the research field as a basis of the decision-making, an in-person meeting is preferable as it makes it easier to address all the outstanding issues, involve people with lived experience, reach consensus and make recommendations by virtue of in-depth, interactive, and nuanced discussions. If people with lived experience attend online, the Chairs need to take a proactive approach to ensure their inclusion in the discussion and a balanced power dynamic. We suggest recording the meetings with designated people for taking minutes, to facilitate a non-biased capture of the complex discussions occurring during triangulation meetings.

### During the triangulation meeting

#### Structure of the meeting

1. The triangulation meeting should start with introductions (including relevant COIs) and a description of the agenda, processes, and roles of the panel members.
2. The *Chair(s)* present the exact purpose of the meeting and what it aims to achieve. The research question should be clearly stated, with a brief reminder about why this question, as well as the studied outcomes, were selected.
3. The *representatives of the evidence* from human and animal data present the results for the LSRs and the SET in a visually efficient way. An initial interpretation could also be presented. The representatives of the evidence answer questions about the data and explain their judgments about the sources and direction of bias in each evidence thesis. Questions might trigger additional analyses, which can be done either on the same day of the meeting or afterwards, depending on the complexity of what is required.
4. Subsequently, *panel members not involved in gathering the data and producing the SET *discuss and interpret the findings. Based on previous work from clinical practice guidelines and our experience applying GATE, we recommend presenting a list of key points to consider, to ensure that discussions stay focused, transparent, and include all the relevant elements for triangulation [8, 50]. The panel will first have to decide whether the evidence from human and animal studies points towards the same direction. If not, the panel will explore possible reasons for the different direction of results, taking into consideration the potential biases in the different strands of evidence. Discussions should also consider whether the conclusion is supported (or not) both by the quantitative evidence from the studies and the qualitative evidence coming from people with lived experience (see Table B, Additional file 1 for a suggested set of considerations when discussing and interpreting data during the triangulation meeting).

An agreement on the outcome of the meeting should be reached about the research question. The answer might not always be binary. Conclusions, rather than being framed as a definitive “effect” or “no effect”, are likely to fall along a continuum. If the different sources of evidence present incompatibilities that the panel considers important, and cannot answer the original research question, several options should be discussed: the original question can be refined, changed or refocused, or the panel can recommend that more data, or data from other sources, need to be gathered in the next iteration of the LSRs (see Table 3, presenting examples from one of GALENOS triangulation meetings).

**Table 3:** Example of triangulation meeting output to answer the question “Do pro-dopaminergic drugs reduce anhedonia in people with major depressive disorder and, if so, to what extent?” [53]

Was the question answered?	Our results are consistent with dopamine playing a role in anhedonia, but do not provide strong evidence that the effect of pro-dopaminergic drugs is greater than that seen in non-dopaminergic drugs. However, the available evidence did not allow us to fully examine the underlying neurobiological framework of anhedonia in humans.
Recommendations for future work and research prioritisation	Recommendations about the design and analysis of future studies on anhedonia in humans and animals HUMANS: • Co-develop and validate rating scales (also with experts in animal models) to investigate translational mechanisms and assess different components of anhedonia in depression • Consider whether pro-dopaminergic drugs might have a role as add-on treatments and whether there are behavioural tasks in humans that are at least phenomenologically closer to the animal work ANIMALS: • Better define the phenotype(s) of depression in animals, in particular its behavioural manifestation • Utilise behavioural assessment tools such as the rat reward learning assay and rodent probabilistic learning tasks and incorporate multiple measures of reward processing • Find and/or use assessment methods which reflect other potential deficits in reward processing more relevant to anhedonia • Strongly encourage pre-registration of animal studies for hypothesis testing animal studies
Recommendations for the next iteration of the living systematic review	• Explore whether the severity of anhedonia at baseline predicts the response to dopaminergic and non-dopaminergic drugs and whether the effect on anhedonia is different to that on depression in general • Evaluate other outcomes related to anhedonia and depression, for instance, anxiety and quality of life

#### Additional issues to consider during the triangulation meeting

It is important that all participants feel comfortable and have time to contribute to the discussion, with particular emphasis on ensuring that people with lived experience are supported throughout the meeting, feel free to speak, are actively engaged in the co-production process and that their perspectives are acknowledged. Having a Co-Chair with a lived experience leadership role can actively support this, for example, using a live online document/chat to bring experiential perspectives into the discussion and serve as a source of written feedback for consideration asynchronously.

As outlined in Step 5, one possible outcome of the meeting may be the decision to collect additional data and defer a final decision to a future iteration of the LSR. However, if an agreed meeting output remains consistently difficult to achieve, multiple triangulation meetings may reach this same conclusion, delaying decision-making indefinitely. We recommend that decisions are not postponed unless the available evidence remains highly ambiguous (a threshold for achieving agreement could be set a priori) and there is a strong likelihood that future data will significantly reduce uncertainty. In such cases, well-defined and specific recommendations about the type and amount of additional data should be made. For example, recommendations might be made about what kind of study designs would be appropriate to answer the question, what kind of outcomes should be included, or what would be the ideal follow-up time.

### After the triangulation meeting

Meeting minutes and action points should be circulated to all participants shortly after the meeting. Additionally, strategies should be developed for communicating the results to relevant stakeholders and translating the meeting output into actionable steps. To maximise the impact and dissemination of the findings, a scientific report—co-authored by the panel members—could be published. Such a paper would comprehensively present the key discussion points and conclusions derived from the triangulation process, facilitating both implementation and knowledge dissemination (see Smith et al., for example [15]).

Finally, we recommend conducting a brief post-meeting survey to gather participants’ feedback and refine both future triangulation meetings for the same question and the triangulation process. We suggest starting with collecting general feedback on the triangulation process and including specific questions that might be relevant to each topic addressed during the meeting.

## Discussion

Based on the limited results from our literature review, our experience from GATE in the GALENOS project, and guidance stemming from clinical practice guidelines, we have illustrated a structured approach for holding efficient meetings to triangulate diverse evidence strands in systematic reviews. Compared with other decision-making processes, such as the development of clinical practice guidelines, there is a lack of guidance available about triangulation meetings and processes, especially in relation to human and animal data. To our knowledge, this is the first attempt to operationalise the process of triangulating data and provide a logical structure within a methodological framework. This paper should serve as a guidance document not only for GALENOS, but also as a template for other projects. These templates will be updated as more LSRs are completed, and we aim to produce a web application that will support researchers across the world by standardising and streamlining the triangulation process.

By cataloguing and evaluating the full spectrum of relevant scientific research including both human and preclinical studies GALENOS will also allow the mental health community of patients, carers, clinicians, researchers and funders to identify better the research questions that most urgently need to be answered [14]. By creating open-access datasets and outputs in a state-of-the-art online resource, GALENOS will help identify promising signals early in the research process [54]. This will accelerate translation from discovery science into effective new interventions for anxiety, depression and psychosis, ready to be translated in clinical practice across the world.

### Limitations

We recognise that this process is specialized and resource-intensive, and that it requires the same people to attend multiple meetings both within the same LSR, and potentially across different systematic reviews. However, this is an investment in terms of saving time and resources, as it allows for identifying and tackling all the potential issues that can lead to delays and misunderstandings within the review team [53]. While the inclusion of people with lived experience is important within our triangulation processes, this is a very specialist topic that requires careful consideration and specific learning. The integration of the perspectives of people with lived experience must always be guided by transparency and critical scrutiny, following the principles of epistemic justice. At present, however, few methodologies exist for systematically interrogating the validity and potential biases within lived experience contributions [55]. We suggest that future developments in triangulation methodology might consider how deliberative dialogue could serve this purpose by surfacing assumptions, exploring tensions, and co-producing interpretations in a rigorous, replicable and methodologically sound manner. Pratt [56] has described three main essential foundations for meaningful engagement of people with lived experience, namely relational, environmental, and personal. Although our approach in GALENOS ensures some of these foundations are met (via training and building trust), future endeavours should take these factors into account in a more structured way. We also acknowledge the need to keep GATE as adaptable and culturally sensitive as possible, for instance, by including in the process people from underprivileged backgrounds and underrepresented populations.

### Future directions

In the future, we will develop training material both on methodology and on triangulation processes for experts with different backgrounds as well as for people with lived experience. We plan to develop software to facilitate the triangulation process and we will also investigate how new technologies, including artificial intelligence tools, could be implemented in GATE. We are working on providing tools focused on assessing the issues of indirectness, risk of bias and reporting quality in animal studies. In terms of co-production, we aim to undertake a systematic evaluation of the co-production processes to generate transferable models for meaningful and equitable co-production in complex global health challenges [16]. Reflections from a person with lived experience have already been published [57] and a paper on the qualitative evaluation of co-production in GALENOS led by members with lived experience is in preparation.

We will test our suggestions in future pre-triangulation and triangulation meetings within the GALENOS project and seek feedback from the participants. In line with the nature of the GALENOS project, this document is also intended to be living and, therefore, adjusted, updated and become more detailed as our experience with holding triangulation meetings is expanding. Although this paper is centred around GATE, the GALENOS project and triangulation of human and animal data, the concepts and processes it has addressed are not restricted to mental health or triangulation of human and animal data and could be applied to other triangulation efforts in different subject areas and with other types of data, and we are open to feedback from other groups.

## Conclusion

Our goal is that this paper and future iterations of this work will contribute to further development of this unique approach for bringing together different types of data, expertise and lived experience in a structured and transparent manner, with the ultimate aim of providing evidence-based research priorities that enable the reduction of research waste and meaningfully advance the field forward [58]. Even though the purpose of the GATE process is not to change clinical practice directly but rather to make recommendations for research prioritisation, this could potentially have an effect in future clinical practice as well, by promoting research for novel treatments that are found to be more effective and eventually accelerating implementation of these treatments in routine clinical care.

## Supplementary Information

Additional File 1. Details on literature searches, PRISMA flow diagram, supplementary tables.

## Data Availability

No datasets were generated or analysed during the current study.

## Abbreviations

GALENOS: Global Alliance for Living Evidence on aNxiety, depressiOn and pSychosis
GATE: GALENOS Approach to Triangulating Evidence
LSR: Living Systematic Review
NTP: National Toxicology Program
IARC: International Agency for Research on Cancer
RoB2: Risk of Bias tool for randomised trials
ROBINS-I: Risk of Bias in Non-randomised Studies of Interventions
SYRCLE: Systematic Review Centre for Laboratory animal Experimentation Risk of Bias tool
GLEAB: Global Lived Experience Advisory Board
COI: Conflict of Interest
SET: Summary of Evidence Tables

## References

[1] World Health Organization, UNAIDS, Global Fund to Fight AIDS T and M. HIV triangulation resource guide: synthesis of results from multiple data sources for evaluation and decision-making. 2009;108.

[2] Noble H, Heale R. Triangulation in research, with examples. Evid Based Nurs. 2019;22:67–8. 10.1136/ebnurs-2019-103145. 31201209 10.1136/ebnurs-2019-103145

[3] Treur JL, Lukas E, Sallis HM, et al. A guide for planning triangulation studies to investigate complex causal questions in behavioural and psychiatric research. Epidemiol Psychiatr Sci. 2024;33:e61. 10.1017/S2045796024000623. 39506622 10.1017/S2045796024000623 PMC7616800

[4] UNAIDS. Monitoring and Evaluation Division. An introduction to triangulation. 2010.

[5] Arias Valencia MM, Principles. Scope, and Limitations of the Methodological Triangulation. Invest Educ Enferm. 2022;40:e03. 10.17533/udea.iee.v40n2e03. 10.17533/udea.iee.v40n2e03 PMC9714985 36264691

[6] Lawlor DA, Tilling K, Davey Smith G. Triangulation in aetiological epidemiology. Int J Epidemiol. 2017;dyw314. 10.1093/ije/dyw314. 10.1093/ije/dyw314 PMC5841843 28108528

[7] Lauer DJ, Russell AJ, Lynch HN, et al. Triangulation of epidemiological evidence and risk of bias evaluation: A proposed framework and applied example using formaldehyde exposure and risk of myeloid leukemias. Global Epidemiol. 2024;7:100143. 10.1016/j.gloepi.2024.100143. 10.1016/j.gloepi.2024.100143 PMC11039339 38659700

[8] Alonso-Coello P, Schünemann HJ, Moberg J et al. GRADE Evidence to Decision (EtD) frameworks: a systematic and transparent approach to making well informed healthcare choices. 1: Introduction. *BMJ*. 2016;i2016. 10.1136/bmj.i2016. 10.1136/bmj.i2016 27353417

[9] Moberg J, Oxman AD, Rosenbaum S, et al. The GRADE Evidence to Decision (EtD) framework for health system and public health decisions. Health Res Policy Syst. 2018;16:45. 10.1186/s12961-018-0320-2. 29843743 10.1186/s12961-018-0320-2 PMC5975536

[10] Alonso-Coello P, Oxman AD, Moberg J et al. GRADE Evidence to Decision (EtD) frameworks: a systematic and transparent approach to making well informed healthcare choices. 2: Clinical practice guidelines. *BMJ*. 2016;i2089. 10.1136/bmj.i2089. 10.1136/bmj.i2089 27365494

[11] Brouwers MC, Kho ME, Browman GP, et al. AGREE II: advancing guideline development, reporting and evaluation in health care. Can Med Assoc J. 2010;182:E839–42. 10.1503/cmaj.090449. 20603348 10.1503/cmaj.090449 PMC3001530

[12] Alonso-Coello P, Irfan A, Sola I, et al. The quality of clinical practice guidelines over the last two decades: a systematic review of guideline appraisal studies. BMJ Qual Saf. 2010;19:e58–58. 10.1136/qshc.2010.042077. 10.1136/qshc.2010.042077 21127089

[13] GALENOS |. Global Alliance for Living Evidence on aNxiety, depressiOn and pSychosis. https://www.galenos.org.uk/. Accessed 29 October 2025.

[14] Cipriani A, Seedat S, Milligan L, et al. New living evidence resource of human and non-human studies for early intervention and research prioritisation in anxiety, depression and psychosis. BMJ Ment Health. 2023;26:e300759. 10.1136/bmjment-2023-300759. 37290906 10.1136/bmjment-2023-300759 PMC10255027

[15] Smith KA, Boyce N, Chevance A, et al. Triangulating evidence from the GALENOS living systematic review on trace amine-associated receptor 1 (TAAR1) agonists in psychosis. Br J Psychiatry. 2024;1–9. 10.1192/bjp.2024.237. 10.1192/bjp.2024.237 39710623

[16] Smith KA, Downs J, Robinson ESJ et al. GALENOS approach to triangulating evidence (GATE): transforming the landscape of psychiatric research. The British Journal of Psychiatry. Published online 2025:1-6. 10.1192/bjp.2025.10457. 10.1192/bjp.2025.10457 PMC7619210 41199459

[17] World Health Organisation. WHO Handbook for Guideline Development. Geneva 2014.

[18] Li S-A, Alexander PE, Reljic T, et al. Evidence to Decision framework provides a structured ‘roadmap’ for making GRADE guidelines recommendations. J Clin Epidemiol. 2018;104:103–12. 10.1016/j.jclinepi.2018.09.007. 30253221 10.1016/j.jclinepi.2018.09.007 PMC7275375

[19] Verkerk K, Van Veenendaal H, Severens JL, et al. Considered judgement in evidence-based guideline development. Int J Qual Health Care. 2006;18:365–9. 10.1093/intqhc/mzl040. 16959800 10.1093/intqhc/mzl040

[20] National Toxicology Program (NTP). National Toxicology Program Cancer Hazard Assessment Report on Night Shift Work and Light at Night. 34197056

[21] IARC Working Group on the Evaluation of Carcinogenic Risks to Humans. Trichloroethylene, tetrachloroethylene, and some other chlorinated agents. Lyon, France: International Agency for Research on Cancer; 2014. PMC4781308 26214861

[22] Pearce N, Blair A, Vineis P, et al. IARC Monographs: 40 Years of Evaluating Carcinogenic Hazards to Humans. Environ Health Perspect. 2015;123:507–14. 10.1289/ehp.1409149. 25712798 10.1289/ehp.1409149 PMC4455595

[23] National Toxicology Program, US Department of Health and Human Services. Handbook for Conducting a Literature-Based Health Assessment Using OHAT Approach for Systematic Review and Evidence Integration. 2019.

[24] Chen VC-H, Hsu T-C, Lin C-F, et al. Association of Risperidone With Gastric Cancer: Triangulation Method From Cell Study, Animal Study, and Cohort Study. Front Pharmacol. 2022;13:846455. 10.3389/fphar.2022.846455. 35444540 10.3389/fphar.2022.846455 PMC9013946

[25] Wiendl H, Gross CC, Bauer J, et al. Fundamental mechanistic insights from rare but paradigmatic neuroimmunological diseases. Nat Rev Neurol. 2021;17:433–47. 10.1038/s41582-021-00496-7. 34050331 10.1038/s41582-021-00496-7

[26] Hindin R, Brugge D, Panikkar B. Teratogenicity of depleted uranium aerosols: A review from an epidemiological perspective. Environ Health. 2005;4:17. 10.1186/1476-069X-4-17. 16124873 10.1186/1476-069X-4-17 PMC1242351

[27] Shapiro DD, Soeung M, Perelli L, et al. Association of High-Intensity Exercise with Renal Medullary Carcinoma in Individuals with Sickle Cell Trait: Clinical Observations and Experimental Animal Studies. Cancers. 2021;13:6022. 10.3390/cancers13236022. 34885132 10.3390/cancers13236022 PMC8656882

[28] Lambraki IA, Majowicz SE, Parmley EJ, et al. Building Social-Ecological System Resilience to Tackle Antimicrobial Resistance Across the One Health Spectrum: Protocol for a Mixed Methods Study. JMIR Res Protoc. 2021;10:e24378. 10.2196/24378. 34110296 10.2196/24378 PMC8262547

[29] Berding K, Vlckova K, Marx W, et al. Diet and the Microbiota–Gut–Brain Axis: Sowing the Seeds of Good Mental Health. Adv Nutr. 2021;12:1239–85. 10.1093/advances/nmaa181. 33693453 10.1093/advances/nmaa181 PMC8321864

[30] Abbas SS, Venkataramanan V, Pathak G, et al. Rabies control initiative in Tamil Nadu, India: a test case for the ‘One Health’ approach. Int Health. 2011;3:231–9. 10.1016/j.inhe.2011.08.001. 24038495 10.1016/j.inhe.2011.08.001

[31] Gómez-Olarte S, Mailänder V, Castro-Neves J, et al. The ENDOMIX perspective: how everyday chemical mixtures impact human health and reproduction by targeting the immune system. Biol Reprod. 2024;111:1170–87. 10.1093/biolre/ioae142. 10.1093/biolre/ioae142 PMC11647104 39446589

[32] Mosimann L, Traoré A, Mauti S, et al. A mixed methods approach to assess animal vaccination programmes: The case of rabies control in Bamako, Mali. Acta Trop. 2017;165:203–15. 10.1016/j.actatropica.2016.10.007. 27751865 10.1016/j.actatropica.2016.10.007

[33] WHO/UNAISDS. Data triangulation workshop Key findings. Tallinn, Estonia 24. May 2011 2011.

[34] Silva M, Loll D, Ezouatchi R, et al. Evaluating a youth-designed sexual and reproductive health mass and social media campaign in Côte d’Ivoire: triangulation of three independent evaluations. Sex Reproductive Health Matters. 2023;31:2248748. 10.1080/26410397.2023.2248748. 10.1080/26410397.2023.2248748 PMC10512782 37728549

[35] HiiL (The Hague Institute for Innovation of Law). Justice Needs and Satisfaction in Fiji. 2018.

[36] JSI. Immunization Data Triangulation Tool (IDTT). 2020.

[37] Ouedraogo O, Siguré S, Sanou S, et al. Landscape analysis of healthcare workforce and resilience actions in the Sahelian context of high security challenge areas: The case of Burkina Faso. Health Plann Manage. 2024;39:933–44. 10.1002/hpm.3764. 10.1002/hpm.3764 38240163

[38] Nyblade L, Stockton MA, Saalim K, et al. Using a mixed-methods approach to adapt an HIV stigma reduction intervention to address intersectional stigma faced by men who have sex with men in Ghana. J Int AIDS Soc. 2022;25:e25908. 10.1002/jia2.25908. 35818873 10.1002/jia2.25908 PMC9274363

[39] Schults JA, Charles KR, Millar J, et al. Establishing a paediatric critical care core quality measure set using a multistakeholder, consensus-driven process. Crit Care Resusc. 2024;26:71–9. 10.1016/j.ccrj.2024.01.002. 39072236 10.1016/j.ccrj.2024.01.002 PMC11282349

[40] Cogliano VJ, Grosse Y, Baan RA, et al. Meeting Report: Summary of *IARC Monographs* on Formaldehyde, 2-Butoxyethanol, and 1- *tert* -Butoxy-2-Propanol. Environ Health Perspect. 2005;113:1205–8. 10.1289/ehp.7542. 16140628 10.1289/ehp.7542 PMC1280402

[41] Daniel Kübler, Low N, Brunold H, Elford J, Hughes G. Rolf Rosenbrock, In collaboration with, Kathrin Frey and Christopher Goodman. Fourth Report of the Surveillance Working Group Federal Commission for Sexual Health. Zurich 2015.

[42] Ostinelli EG, Chiocchia V, Macleod M, et al. Pro-dopaminergic pharmacological interventions for anhedonia in depression: protocol for a living systematic review of human and non-human studies. Wellcome Open Res. 2023;8:425. 10.12688/wellcomeopenres.19870.1. 39026608 10.12688/wellcomeopenres.19870.1 PMC11255544

[43] Methodology | GALENOS. https://www.galenos.org.uk/methodology. Accessed 29 October 2025.

[44] Chiocchia V, Higgins JPT, Siafis S, et al. A framework for collating and synthesizing evidence from human and animal studies: the GALENOS approach. *In review*.

[45] Sterne JAC, Savović J, Page MJ, et al. RoB 2: a revised tool for assessing risk of bias in randomised trials. BMJ. 2019;l4898. 10.1136/bmj.l4898. 10.1136/bmj.l4898 31462531

[46] Sterne JA, Hernán MA, Reeves BC, et al. ROBINS-I: a tool for assessing risk of bias in non-randomised studies of interventions. BMJ. 2016;i4919. 10.1136/bmj.i4919. 10.1136/bmj.i4919 PMC5062054 27733354

[47] Hooijmans CR, Rovers MM, De Vries RB, et al. SYRCLE’s risk of bias tool for animal studies. BMC Med Res Methodol. 2014;14:43. 10.1186/1471-2288-14-43. 24667063 10.1186/1471-2288-14-43 PMC4230647

[48] Sunkel C, Sartor C. Perspectives: involving persons with lived experience of mental health conditions in service delivery, development and leadership. BJPsych Bull. 2022;46:160–4. 10.1192/bjb.2021.51. 33977895 10.1192/bjb.2021.51 PMC9346508

[49] Hanson-DeFusco J. What data counts in policymaking and programming evaluation – Relevant data sources for triangulation according to main epistemologies and philosophies within social science. Eval Program Plan. 2023;97:102238. 10.1016/j.evalprogplan.2023.102238. 10.1016/j.evalprogplan.2023.102238 36680973

[50] Rutherford GW, McFarland W, Spindler H, et al. Public health triangulation: approach and application to synthesizing data to understand national and local HIV epidemics. BMC Public Health. 2010;10:447. 10.1186/1471-2458-10-447. 20670448 10.1186/1471-2458-10-447 PMC2920890

[51] Morley R, Gilbert D. for the GALENOS Work Package 1 Team. GALENOS Involvement of People with Lived Experience in Living Systematic Reviews. 2023.

[52] MQ Mental Health Research | Transforming The Future. of Mental Illness. https://www.mqmentalhealth.org/home/. Accessed 29 October 2025.

[53] Ostinelli E, Salanti G, Macleod M, et al. Pro-dopaminergic pharmacological interventions for anhedonia in depression: a living systematic review and network meta-analysis of human and animal studies. eBioMedicine. 2025;121. 10.1016/j.ebiom.2025.105967 PMC12790147 41168072

[54] Hastings J, Wosny M, Kennett J, et al. Automatically detecting trends and open questions from mental health publications: a Wellcome-funded GALENOS project. BMJ Ment Health. 2026;29:e302379. 10.1136/bmjment-2025-302379. 41927103 10.1136/bmjment-2025-302379 PMC13055325

[55] Jazayeri D, Banfield M, Tapp C, et al. Capacity-building strategy for next-generation mental health research: embedding a national network infrastructure to grow mental health researcher capabilities and mental health lived-experience research leaders. BMJ Ment Health. 2025;28:e301554. 10.1136/bmjment-2025-301554. 40132904 10.1136/bmjment-2025-301554 PMC11938221

[56] Pratt B. Achieving inclusive research priority-setting: what do people with lived experience and the public think is essential? BMC Med Ethics. 2021;22:117. 10.1186/s12910-021-00685-5. 34481506 10.1186/s12910-021-00685-5 PMC8418727

[57] Downs J. Beyond the methodological binary: coproduction as the third pillar of mental health science. BMJ Ment Health. 2025;28:e301807. 10.1136/bmjment-2025-301807. 40930753 10.1136/bmjment-2025-301807 PMC12421632

[58] Malhi GS, Marsh J, Ongur D, et al. Truthful communication of mental science: pledge to our patients and profession. Br J Psychiatry. 2025;226:253–5. 10.1192/bjp.2025.94. 40268759 10.1192/bjp.2025.94

