# Supplementary material for: The GALENOS approach to triangulating evidence: a structured approach for integrating information from human and animal studies
Source: BMC Med Res Methodol. 2026 Jun 2;26:169. doi: 10.1186/s12874-026-02891-4 (PMC13445838; doi:10.1186/s12874-026-02891-4)
Supplement: Supplementary file 1 [file 12874_2026_2891_MOESM1_ESM.docx]

**The GALENOS Approach to Triangulating Evidence: A structured approach for integrating information from human and animal studies.**

**Additional file 1**

**Details on the literature searches:**

We identified 254 potentially relevant documents. After title/abstract and full-text screening, we included 11 documents describing data triangulation from human and animal studies (1–11) (Figure A), including one document that we identified from an unsystematic search (11) (Appendix, Table A). In nine of these papers, it was not clear whether a triangulation framework was used, or when used, it was often not described in detail. These nine papers present triangulation findings narratively, without sufficient detail on how the conclusions were derived. Only two documents provide a detailed description of the triangulation framework, published by the National Toxicology Program (NTP) and the International Agency for Research on Cancer (IARC) (3,11). Additional triangulation frameworks relevant to causal and aetiological research questions—largely theoretical but including some practical examples—have also been published (12–15).

The second literature search identified 13 documented triangulation meetings (irrespective of their inclusion of animal data) (16–28). Of these, two were referring to GALENOS-related meetings (18,28). The remaining eleven referred to the triangulation of different types of human data, but contained little information on how the triangulation meetings were structured or conducted.

**Figure A: PRISMA flow diagram**

**Identification of studies via databases and registers**

Records removed *before screening*:

(n = 0)

Records identified from:

Databases (n = 254)

**Identification**

Records screened

(n = 254)

Records excluded

(n = 223)

Reports sought for retrieval

(n = 31)

Reports not retrieved

(n = 0)

**Screening**

Reports assessed for eligibility

(n = 31)

Reports excluded (n=21)

Human data only (n = 4)

No triangulation (n =14)

Duplicate (n=1)

Review (n=2)

Studies included in review

(n = 11)

Studies identified from other sources and included

(n =1)

**Included**

**Table A: Characteristics of included documents that explicitly describe triangulation of human and animal studies as a primary aim or focus.**

| **Author, Year, Paper Title**  **DOI** | **Types of data triangulated** | **Triangulation framework described/used?** | **Triangulation meeting held/described?** |
| --- | --- | --- | --- |
| Chen (2022): *Association of Risperidone With Gastric Cancer: Triangulation Method From Cell Study, Animal Study, and Cohort Study*  10.3389/fphar.2022.846455 | In vitro and animal experiments and a retrospective cohort study in humans | No: no framework described and no details on how triangulation was achieved in this particular case | Not reported |
| Wiendl (2021*) Fundamental mechanistic insights from rare but paradigmatic neuroimmunological diseases*  10.1038/s41582-021-00496-7 | Human and animal data | Yes: no details on how triangulation was achieved in this particular case | Not reported |
| National Toxicology Program (2021*) NTP Cancer Hazard Assessment Report on Night Shift Work and Light at Night*  10.22427/NTP-CHR-NSWLAN | Cancer epidemiological and mechanistic studies in humans, animals and in vitro experiments | Yes: detailed description of how triangulation was achieved in this particular case | Yes, not described in detail |
| Hindin (2005) *Teratogenicity of depleted uranium aerosols: a review from an epidemiological perspective*  10.1186/1476-069X-4-17 | Human epidemiological data are reviewed in this paper; however, animal data are taken into consideration for the triangulation | Yes; no details on how triangulation was achieved in this particular case | Not reported |
| Shapiro (2021) *Association of High-Intensity Exercise with Renal Medullary Carcinoma in Individuals with Sickle Cell Trait: Clinical Observations and Experimental Animal Studies*  10.3390/cancers13236022 | Human and animal data | No; no framework described and no details on how triangulation was achieved in this particular case | Not reported |
| Lambraki (2021) *Building Social-Ecological System Resilience to Tackle Antimicrobial Resistance Across the One Health Spectrum: Protocol for a Mixed Methods Study*  10.2196/24378 | Human and animal data | No; no framework described, but some description on how triangulation will be achieved during workshops | Planned and described in detail |
| Berding (2021) *Diet and the Microbiota-Gut-Brain Axis: Sowing the Seeds of Good Mental Health*  10.1093/advances/nmaa181 | Human, animal and in vitro | No framework described; no details on how triangulation was achieved in this particular case | Not reported |
| Abbas (2011) *Rabies control initiative in Tamil Nadu, India: A test case for the 'One Health' approach*  10.1016/j.inhe.2011.08.001 | Human and animal  (Surveillance data on dog bites, vaccine consumption data and dog population data) | Yes; no detailed description of how triangulation was achieved in this particular case | Not clear but data were shared with stakeholders for comments |
| *IARC Monographs on the Evaluation of Carcinogenic Risks to Humans Volume (2014)*  *Trichloroethylene, Tetrachloroethylene, and Some Other Chlorinated Agents*  *ISBN-13*  *978-92-832-0144-1* | Cancer studies in human and animals and in vitro data | Yes; detailed description of how triangulation was achieved in this particular case | Yes; general description of meeting processes in IARC, but not for this particular case |
| *Gomez-Olarte (2024) The ENDOMIX perspective: how everyday chemical mixtures impact human health and reproduction by targeting the immune system**  *10.1093/biolre/ioae142*  *** *Presentation of the approach; the ENDOMIX project itself is currently undergoing* | Human cohort, animal data and in vitro data | Yes; briefly described | No |
| *Mosimann (2016) A mixed methods approach to assess animal vaccination programmes: The case of rabies control in Bamako, Mali*  *10.1016/j.actatropica.2016.10.007* | Human and animal data (Tracking of animals, animal vaccination coverage data, household survey, questionnaire, spatial analysis, observations) | Yes | No |

**Box A: Uncertainties discussed during the pre-triangulation meeting for the LSR on circadian disruption and mood disorders, and their resolution in the protocol.**

| **Review Questions**   - Is there evidence that supports the existence of a causal relationship between circadian disruption and mood disorders? - If this causal relationship is demonstrated, how do social and cultural factors interact with circadian disruption and mood disorders? | |
| --- | --- |
| **Uncertainties** | **Refinements in the protocol** |
| ***Definition of the question*** | |
| **Questions need to be refocused** | The first iteration of the review will focus on the question of causality. If evidence of causality is found, next iterations will possibly assess the role of circadian disruption as a potential mediator. |
| ***Definition of circadian disruption*** | |
| **Definition of circadian disruption needs to be more specific**  (e.g. duration of disruption, types of disorders that should be included or not) | Circadian disruption will be included if it has been present for 3 months or more.  Sleep disorders alone will not be included, unless they are part of the symptomatology of circadian disruption.  Jet lag and disorders will not be included in the first iteration of the review due to their transient nature.  To address delayed or advanced sleep-wake disorder, for this iteration, a group of experts (including experts with lived experience) will be convened to consider the evidence from each individual study (including the use or not of standard rating scales such as the Morningness-Eveningness Questionnaire (MEQ)) and advise whether these should be included as examples of circadian disruption. |
| ***Definition of outcomes*** | |
| **Mood symptoms:**  Outcome needs refinement, for example will we include only clinical diagnoses of a mood disorder? | Mood symptoms will include not only a clinical diagnosis of mood disorder (for example major depression) but also individual mood symptoms which contribute to a diagnosis of mood disorder (such as low mood, weight change, anhedonia). |
| ***Study design*** | |
| **Which study design should we include?**  **Will we include intervention studies in mood disorders?** | For circadian disruption as exposure, it is not possible to have RCTs, even if, in theory, they would offer the highest quality of evidence. We will, therefore, include all types of observational studies focussing on the relationship between circadian disruption and mood disorders.  We will also include RCTs in mood disorders testing an intervention aimed at modifying circadian disruption (such as increased daytime light exposure, night-time use of melatonin). In the first iteration of the LSR we will focus only on RCTs but may include other study designs in future iterations. |
| ***Study selection*** | |
| **What to do with sequential studies?** | Authors will be contacted to clarify how many of the repeated measures were in the same or different populations. |

**Table B: Suggested framework for discussing evidence from human and animal studies in GATE**

| **Question** | **Findings** | **Additional considerations** |
| --- | --- | --- |
| What is the evidence from human data? |  |  |
| What is the risk and direction of bias in the evidence of human data? |  |  |
| What is the evidence from animal data? |  |  |
| What is the risk and direction of bias in the evidence from animal data? |  |  |
| What are the most important considerations made by experts with lived experience? |  |  |
| Are the results of human and in-vivo data pointing to the same direction? |  |  |
| If not, what are some possible reasons for the different direction? |  |  |
| For questions on causality, which hypotheses hold up to the maximum criteria for causality and the most important criteria for causality? |  |  |
| Was the original research question answered by both quantitative and qualitative (lived experience, clinical experience, mechanistic information) data? |  |  |
| Has the question been adequately answered? |  |  |
| If the research question has not been adequately answered, does the research question need to be refined/changed/refocused? |  |  |
| If not, would more data help us answering the question? |  |  |
| If yes, what kind of data would make it possible to answer this question? |  |  |
| Would it be feasible to obtain such data in a reasonable timeframe? |  |  |

1.	Chen VCH, Hsu TC, Lin CF, Huang JY, Chen YL, Tzang BS, et al. Association of Risperidone With Gastric Cancer: Triangulation Method From Cell Study, Animal Study, and Cohort Study. Front Pharmacol. 2022 Apr 4;13:846455.

2.	Wiendl H, Gross CC, Bauer J, Merkler D, Prat A, Liblau R. Fundamental mechanistic insights from rare but paradigmatic neuroimmunological diseases. Nat Rev Neurol. 2021 Jul;17(7):433–47.

3.	National Toxicology Program (NTP). National Toxicology Program Cancer Hazard Assessment Report on Night Shift Work and Light at Night.

4.	Hindin R, Brugge D, Panikkar B. Teratogenicity of depleted uranium aerosols: A review from an epidemiological perspective. Environ Health. 2005 Dec;4(1):17.

5.	Shapiro DD, Soeung M, Perelli L, Dondossola E, Surasi DS, Tripathi DN, et al. Association of High-Intensity Exercise with Renal Medullary Carcinoma in Individuals with Sickle Cell Trait: Clinical Observations and Experimental Animal Studies. Cancers. 2021 Nov 30;13(23):6022.

6.	Lambraki IA, Majowicz SE, Parmley EJ, Wernli D, Léger A, Graells T, et al. Building Social-Ecological System Resilience to Tackle Antimicrobial Resistance Across the One Health Spectrum: Protocol for a Mixed Methods Study. JMIR Res Protoc. 2021 Jun 10;10(6):e24378.

7.	Berding K, Vlckova K, Marx W, Schellekens H, Stanton C, Clarke G, et al. Diet and the Microbiota–Gut–Brain Axis: Sowing the Seeds of Good Mental Health. Adv Nutr. 2021 Jul;12(4):1239–85.

8.	Abbas SS, Venkataramanan V, Pathak G, Kakkar M. Rabies control initiative in Tamil Nadu, India: a test case for the ‘One Health’ approach. Int Health. 2011 Dec;3(4):231–9.

9.	Gómez-Olarte S, Mailänder V, Castro-Neves J, Stojanovska V, Schumacher A, Meyer N, et al. The ENDOMIX perspective: how everyday chemical mixtures impact human health and reproduction by targeting the immune system. Biol Reprod. 2024 Dec 12;111(6):1170–87.

10.	Mosimann L, Traoré A, Mauti S, Léchenne M, Obrist B, Véron R, et al. A mixed methods approach to assess animal vaccination programmes: The case of rabies control in Bamako, Mali. Acta Trop. 2017 Jan;165:203–15.

11.	IARC Working Group on the Evaluation of Carcinogenic Risks to Humans. Trichloroethylene, tetrachloroethylene, and some other chlorinated agents. Lyon, France: International Agency for Research on Cancer; 2014. 1 p. (IARC monographs on the evaluation of carcinogenic risks to humans).

12.	Treur JL, Lukas E, Sallis HM, Wootton RE. A guide for planning triangulation studies to investigate complex causal questions in behavioural and psychiatric research. Epidemiol Psychiatr Sci. 2024;33:e61.

13.	Lawlor DA, Tilling K, Davey Smith G. Triangulation in aetiological epidemiology. Int J Epidemiol. 2017 Jan 20;dyw314.

14.	Lauer DJ, Russell AJ, Lynch HN, Thompson WJ, Mundt KA, Checkoway H. Triangulation of epidemiological evidence and risk of bias evaluation: A proposed framework and applied example using formaldehyde exposure and risk of myeloid leukemias. Glob Epidemiol. 2024 Jun;7:100143.

15.	Steenland K, Schubauer-Berigan MK, Vermeulen R, Lunn RM, Straif K, Zahm S, et al. Risk of Bias Assessments and Evidence Syntheses for Observational Epidemiologic Studies of Environmental and Occupational Exposures: Strengths and Limitations. Environ Health Perspect. 2020 Sep;128(9):095002.

16.	World Health Organization, UNAIDS, Global Fund to Fight AIDS T and M. HIV triangulation resource guide : synthesis of results from multiple data sources for evaluation and decision-making. 2009;108.

17.	Noble H, Heale R. Triangulation in research, with examples. Evid Based Nurs. 2019 Jul;22(3):67–8.

18.	Smith KA, Boyce N, Chevance A, Chiocchia V, Correll CU, Donoghue K, et al. Triangulating evidence from the GALENOS living systematic review on trace amine-associated receptor 1 (TAAR1) agonists in psychosis. Br J Psychiatry. 2024 Dec 23;1–9.

19.	WHO/UNAISDS. Data triangulation workshop Key findings [Internet]. Tallinn, Estonia 24 May 2011; 2011. Available from: https://iris.who.int/bitstream/handle/10665/370570/WHO-EURO-2011-6462-46228-66877-eng.pdf?sequence=1&isAllowed=y

20.	Silva M, Loll D, Ezouatchi R, Kassegne S, Nagbe RHY, Babogou L, et al. Evaluating a youth-designed sexual and reproductive health mass and social media campaign in Côte d’Ivoire: triangulation of three independent evaluations. Sex Reprod Health Matters. 2023 Dec 31;31(1):2248748.

21.	HiiL (The Hague Institute for Innovation of Law). Justice Needs and Satisfaction in Fiji. 2018.

22.	JSI. Immunization Data Triangulation Tool (IDTT) [Internet]. 2020. Available from: https://uifhs.jsi.com/wp-content/uploads/2020/09/Immunization-data-triangulation-tool_User-guide_v08.31.20.pdf

23.	Ouedraogo O, Siguré S, Sanou S, Kaboré A, Dahany MM, Diallo S. Landscape analysis of healthcare workforce and resilience actions in the Sahelian context of high security challenge areas: The case of Burkina Faso. Int J Health Plann Manage. 2024 May;39(3):933–44.

24.	Nyblade L, Stockton MA, Saalim K, Rabiu Abu‐Ba’are G, Clay S, Chonta M, et al. Using a mixed‐methods approach to adapt an HIV stigma reduction intervention to address intersectional stigma faced by men who have sex with men in Ghana. J Int AIDS Soc. 2022 Jul;25(S1):e25908.

25.	Schults JA, Charles KR, Millar J, Rickard CM, Chopra V, Lake A, et al. Establishing a paediatric critical care core quality measure set using a multistakeholder, consensus-driven process. Crit Care Resusc. 2024 Jun;26(2):71–9.

26.	Cogliano VJ, Grosse Y, Baan RA, Straif K, Secretan MB, Ghissassi FE, et al. Meeting Report: Summary of *IARC Monographs* on Formaldehyde, 2-Butoxyethanol, and 1- *tert* -Butoxy-2-Propanol. Environ Health Perspect. 2005 Sep;113(9):1205–8.

27.	Daniel Kübler, Nicola Low, Herbert Brunold, Jonathan Elford, Gwenda Hughes, Rolf Rosenbrock, In collaboration with, Kathrin Frey and Christopher Goodman. Fourth Report of the Surveillance Working Group Federal Commission for Sexual Health. Zurich; 2015.

28.	Ostinelli EG, Chiocchia V, Macleod M, Browning M, Harmer C, Siafis S, et al. Pro-dopaminergic pharmacological interventions for anhedonia in depression: protocol for a living systematic review of human and non-human studies. Wellcome Open Res. 2023 Oct 5;8:425.
